# Transgenerational Effects of Parental Larval Diet on Offspring Development Time, Adult Body Size and Pathogen Resistance in *Drosophila melanogaster*

**DOI:** 10.1371/journal.pone.0031611

**Published:** 2012-02-16

**Authors:** Terhi M. Valtonen, Katariina Kangassalo, Mari Pölkki, Markus J. Rantala

**Affiliations:** Department of Biology, Section of Ecology, University of Turku, Turku, Finland; University of Otago, New Zealand

## Abstract

Environmental conditions experienced by parents are increasingly recognized to affect offspring performance. We set out to investigate the effect of parental larval diet on offspring development time, adult body size and adult resistance to the bacterium *Serratia marcescens* in *Drosophila melanogaster*. Flies for the parental generation were raised on either poor or standard diet and then mated in the four possible sex-by-parental diet crosses. Females that were raised on poor food produced larger offspring than females that were raised on standard food. Furthermore, male progeny sired by fathers that were raised on poor food were larger than male progeny sired by males raised on standard food. Development times were shortest for offspring whose one parent (mother or the father) was raised on standard and the other parent on poor food and longest for offspring whose parents both were raised on poor food. No evidence for transgenerational effects of parental diet on offspring disease resistance was found. Although paternal effects have been previously demonstrated in *D. melanogaster*, no earlier studies have investigated male-mediated transgenerational effects of diet in this species. The results highlight the importance of not only considering the relative contribution each parental sex has on progeny performance but also the combined effects that the two sexes may have on offspring performance.

## Introduction

Since phenotypic development is the result of a complex interplay between the genetic architecture of an organism and the environment it experiences during development, a given genotype can give rise to a variety of phenotypes depending on the environmental conditions [1]. In addition to direct environmental effects current and past environmental conditions experienced by other individuals, often the parent(s), may be important in shaping an organism's phenotype [2]. In fact, it has been suggested that past environmental circumstances may contribute as much as present conditions to variation in current performance [3]. Parental effect is defined as any effect on offspring phenotype that is not determined by the offspring's DNA but instead is brought about by the genotype or environmental experience of its parents [4], [5]. Parents that acquire high condition from a resource-rich environment may benefit by transferring their condition to their offspring, which due to their higher quality will do better under any environmental conditions than offspring of poor-quality parents [2], [6]. On the other hand, parents may also respond to environmental cues in ways that enhance offspring performance under particular environmental circumstances. Under this scenario, offspring will do best in an environment similar to that experienced by their parents [2], [7].

Variation in parental nutrient provisioning is considered particularly important in shaping offspring phenotype [4]. Whereas diet restriction and mild starvation are often associated with increased longevity and stress tolerance [8]–[11] poor nutrition during early development is generally associated with negative effects on many adult traits such as body size, survival, secondary sexual trait expression, stress and disease resistance [12]–[20]. Even if a malnourished individual appears to recover from the nutritional deprivation when food conditions improve, nutritional deficits experienced during key periods of development may have permanent effects on the adult individual and even on its offspring [6], [13], [21], [22]. The complex effect of diet on individual performance is further demonstrated by the growing number of studies showing interaction between parental and offspring nutrition in their effect on offspring performance [6], [17], [22]–[26]. Maternal effects are typically considered more important than paternal effects due to the tendency for mothers to invest more resources in production and care of offspring [2], [27]–[30]. The effect of maternal nutrient provisioning on offspring condition and life-history has been documented for a number of species including many insects [2], [4], [31], [32]. Although paternal effects have been reported in species where males contribute to offspring care or provide females with nutrition or other substances that can be transferred to eggs/embryos by the female [4], [32]–[40] parental effects are often assumed to be mediated solely by the mother when males do not partake in progeny care in the conventional sense [24], [40], [41]. Recent studies showing transgenerational epigenetic effects have however started to question the relevance of this assumption [42], [43].

One such species where males make no obvious material contribution to offspring is *Drosophila melanogaster* [44]. Even though it is used extensively for studies of nutrition-related life-history trade-offs relatively little is known about cross-generational dietary effects in this species [23]. Whereas maternal dietary effects have been previously described in *D. melanogaster* [6], [23] no data exist for paternal dietary effects. In *D. melanogaster*, several studies have described paternal effects of temperature and light regimes on a variety of traits including development time and density sensitivity [45], early fecundity [46], cold tolerance [47] and egg size [48]. Furthermore, in a recently published paper by Friberg et al. [43] substantial variation in egg-to-adult survival owing to paternal effects was uncovered in this species. Transgenerational epigenetic effects were suggested as the most feasible candidate for the observed paternal effects. In mice and in the fly *Telostylinus angusticollis* dietary effects of both mothers and fathers have been shown to be transmissible to the next generation [24], [41]. Because only a few studies have actually tested for environmentally induced paternal effects in species that lack direct paternal investment, the effect of the paternal environment or the potential for joint effects of both parental environments on offspring performance remain poorly understood in such species [24].

In vertebrates offspring can inherit maternal immune function through antibodies [49]. Similar phenomena have recently been observed among invertebrates that rely solely on innate immunity for defense against infection [50]–[53]. In transgenerational immunity, both the mother and her environment have been shown to influence the phenotype of the offspring. For example, female *Daphnia* that reproduced under poor nutritional conditions were found to produce offspring that were more resistant to a bacterial pathogen than offspring of mothers that reproduced in a high-food environment [54]. In invertebrates, studies on trans-generational priming have thus far focused mainly on a transfer via the mother. Using the red flour beetle, *Tribolium castaneum*, Roth et al. [55] challenged the traditional view that males provide only genes to their offspring in species without parental care by demonstrating that trans-generational immune priming can occur also through fathers. Transgenerational effects of nutrition on disease susceptibility are rather well acknowledged among vertebrates. The ways in which invertebrate offspring resistance relates to aspects of parental experience other than pathogen pre-exposure have not been systemically investigated [17], [26], [56].

In the present study we aimed to detect any transgenerational effects of parental early nutrition (poor vs. standard) on offspring development time, adult body size and adult susceptibility to the bacterium *Serratia marcescens* in *Drosophila melanogaster*. We only manipulated the parental larval diet, with all adults being placed on standard food on the day they emerged from their puparia. We tested for both maternal and paternal dietary effects as well as for their interaction on offspring raised themselves under standard nutritional conditions. The results of the present study demonstrate the importance of not only considering the relative contribution each parental sex has on progeny performance but also the combined effects that the two sexes may have on offspring performance.

## Materials and Methods

### Flies and husbandry

Flies (*D. melanogaster*) used in the experiment were collected from a laboratory base population that had been maintained in the laboratory at room temperature (23±1°C) for approximately four years before the study commenced. Stock larvae are reared on: 10 g agar, 80 g cornmeal, 20 g brewer's yeast, 1.5 dl syrup, 10 ml nipagin, 1 L water diet (henceforth referred as standard food/standard recipe) and adult flies are fed baker's yeast. The stock originates from approximately 500 females collected by baits from an apple grove at Lappi in Southern Finland in September 2006. Since their establishment in the laboratory the stock has been expanded and maintained in large glass jars with a standing adult population of several thousand individuals.

### Breeding design and development time

For the present study several hundred individuals (400 ♂, 400 ♀) were collected as virgin from the stock. At the age of 4–5 days post eclosion the flies were released in a cage and allowed to mate and lay eggs on baker's yeast supplemented petri dishes for 24 hours. The following day eggs were harvested and transferred either into ‘standard food’ or ‘poor food’ vials at a density of 20 eggs per vial (altogether 50 vials per condition). The ‘standard food’ vials contained 15 ml standard food for the larvae. The ‘poor food’ vials also contained 15 ml standard food but the amount of brewer's yeast was reduced to 1/8 of the standard amount [19], [57]–[59]. The vials were maintained at 22°C in a 12 L: 12D light regime. As adults emerged (parental generation) they were collected as virgins, housed in same sex groups of 5–8 individuals in vials supplemented with baker's yeast and at the age of 4–5 days post eclosion crossed in the four possible sex-by-developmental nutrition combinations:

males on standard food×females on standard food (S-S, 75 pairs)
males on poor food×females on poor food (P-P, 74 pairs)
males on standard food×females on poor food (S-P, 72 pairs)
males on poor food×females on standard food (P-S, 75 pairs).

The pairs were allowed to interact and lay eggs for 24 hours in 30 ml vials supplemented with baker's yeast to enhance egg laying (one pair in each vial). The following day eggs were harvested and transferred into ‘standard food’ vials at a density of 20 eggs per vial (20 eggs from each pair in one vial) and placed at 22°C in a 12 L: 12D light regime. Development time of the next generation flies was measured as the length of time between oviposition and adult eclosion. To measure the development time the emerged adults were collected three times a day until eclosion ceased. To ensure virginity, the flies were collected as virgin and housed in same sex groups of 5–8 individuals in vials supplemented with baker's yeast. Half of the adult flies in each vial were subsequently assigned for the immunity assay; the other half was used as a control (see below). Ice and CO_2_ were used in handling the flies.

### Pathogen resistance

In the immunity assay survival against a Gram-negative entomopathogenic bacterium *Serratia marcescens* was measured. The immunity assay was performed on adult flies aged between 5–7 days (post eclosion) and it was carried out in three sets during three successive days (henceforth referred to as experiment day). To measure the strength of immunity towards the bacterium, flies were anesthetized with CO_2_, placed on ice, and the thoraces of individual flies pierced with a 0.1 mm pin dipped in a suspension of an overnight culture of the bacteria in liquid broth (OD_590_ = 0.039, LB = 10 g tryptone, 5 g yeast extract and 10 g NaCl, 1 L water). After infection, the flies (females: n_(S-S)_ = 271, n_(P-P)_ = 218, n_(S-P)_ = 238, n_(P-S)_ = 219; males: n_(S-S)_ = 244, n_(P-P)_ = 157, n_(S-P)_ = 230, n_(P-S)_ = 207) were placed on fresh food and housed in same sex groups of 2–5 individuals at room temperature (23±1°C). Our previous studies [59] have shown that control flies only pricked with a pin dipped in liquid broth (10 g tryptone, 5 g yeast extract and 10 g NaCl, 1 L water) survive the assay period and hence, in this experiment the control flies (females: n_(S-S)_ = 278, n_(P-P)_ = 228, n_(S-P)_ = 245, n_(P-S)_ = 231; males: n_(S-S)_ = 244, n_(P-P)_ = 164, n_(S-P)_ = 234, n_(P-S)_ = 208) were only transferred into fresh food vials. The survival of the flies was scored twice daily. Individuals that survived five days were considered to have survived the treatment. The outline of the bacterial infection follows the assay used by Lazzaro et al. [60], [61] and Valtonen et al. [59], [62].

### Adult size

Flies that were used in the immunity assay as control flies as well as those extra individuals that were reserved for the immunity assay but that were not needed in the assay after all were subsequently assigned for the body size assay. Adult body size (thorax length) was measured under a light microscope using an ocular micrometer. Because a large portion of the flies that did not survive the bacterial infection were too fragile to be handled the infected group of individuals was not measured (neither the ones that survived the infection nor the ones that did not) and hence, we do not have size data for the infected group of flies. However, because the flies were randomly assigned for either the bacterial exposure or the control group (see above) and because of the rather large number of measured flies (altogether 895 females, 794 males) we can be fairly confident that the data gives a realistic picture of the size distribution among the flies in general.

### Statistical methods

Prior to statistical analysis an average offspring body size and an average offspring development time was calculated for each parental pair (i.e. vial means for males and females) to avoid pseudoreplication. The effect of parental diet on offspring size (vial means) was analyzed using the univariate analysis of variance, with maternal diet, paternal diet and sex as fixed factors and rearing vial as a random effect.

Development time was analyzed using Cox regression survival analysis (Cox proportional hazards regression). The main effects maternal diet, paternal diet and sex and all possible two-and three-way interactions terms between these variables were included as covariates in the model. In the model maternal diet, paternal diet and sex were presented as categorical covariates and development time (vial means) as the dependent variable. The significance of the model variables were estimated using the simultaneous method. Kaplan-Mayer survival analysis was used for the multiple comparisons (reduced probability value of P = 0.05/6 = 0.008 was used to control for multiple comparisons).

Binary logistic regression analysis was used to identify factors associated with pathogen resistance. Survival, which is a binary variable (i.e. takes the value 0 or 1), was set as the dependent variable. The main effects: parental pair (i.e. rearing vial), maternal diet, paternal diet, sex, experiment day and treatment, two-way interactions: maternal diet×treatment, paternal diet×treatment, sex×treatment, three-way interactions: maternal diet×paternal diet×treatment, maternal diet×sex×treatment, paternal diet×sex×treatment and a four-way interaction: maternal diet×paternal diet×sex×treatment were included as covariates in the model. The significance of the model variables were estimated using the simultaneous method and the performance of the model was statistically evaluated using the Hosmer-Lemeshow goodness-of-fit test. All statistics were conducted using IBM SPSS Statistics 19 for Windows.

## Results

### Adult size

**Figure 1:**
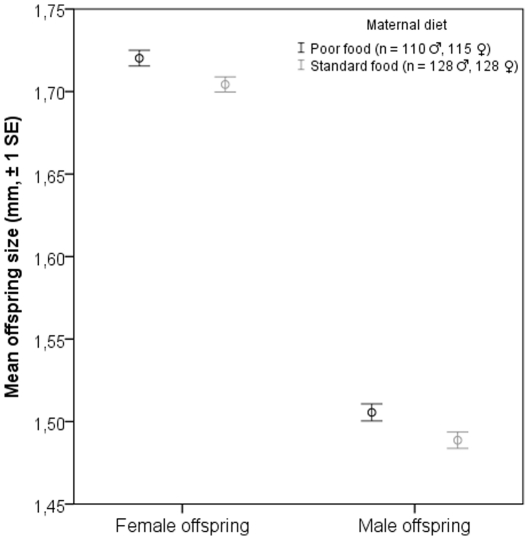
Mean body size (thorax length) of female and male offspring. Females raised on a poor diet produced larger offspring than females that were raised on a standard diet.

**Figure 2:**
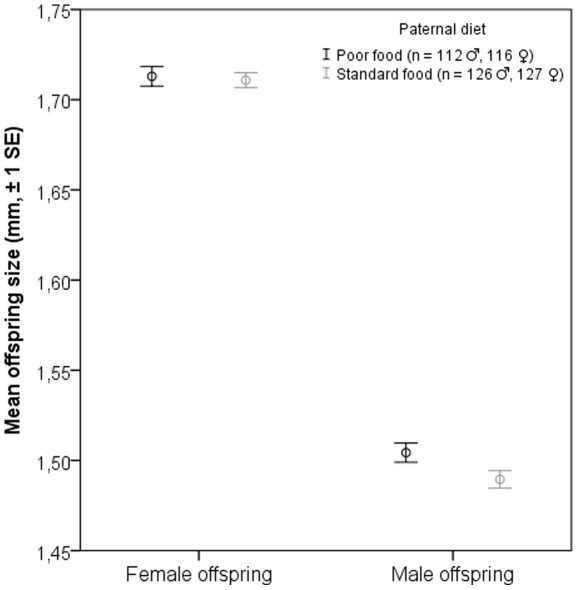
Mean body size (thorax length) of female and male offspring. Males raised on a poor diet produced larger sons than males raised on a standard diet. No effect of paternal diet on female body size was detected.

Sex and maternal diet had a statistically significant effect on offspring body size. The effect of rearing vial, treated as a random factor, was also significant and the paternal diet×sex interaction was marginally significant (Table 1). To elucidate the meaning of the interaction term, ANOVA was run again, but this time separately for males and females with maternal diet and paternal diet as fixed factors. Whereas a statistically significant effect of maternal diet on adult body size was found in both male and female offspring, a statistically significant paternal effect on body size was detected only among male offspring (Table 2). According to the results females were larger than males. Females raised on poor diet produced larger offspring than females that were raised on standard diet (Figure 1). Accordingly, males raised on poor diet sired larger sons than males that were raised on standard diet (Figure 2).

**Table 1:** Summary of analysis of variance on offspring body size (thorax length) with data pooled over sexes. Significant effects are shown in bold.

	df	Type I SS	MS	F	P
**Maternal diet**	**1**	**0.042**	**0.042**	**10.34** *	**0.001**
Paternal diet	1	0.010	0.010	2.39**	0.124
**Sex**	**1**	**5.564**	**5.564**	**3452.03** †	**<0.001**
Maternal diet×Paternal diet	1	0.007	0.007	1.83††	0.178
Maternal diet×Sex	1	6×10^−5^	6×10^−5^	0.04‡	0.848
Paternal diet×Sex	1	0.006	0.006	3.45‡‡	0.064
Maternal diet×Paternal diet×Sex	1	0.001	0.001	0.74+	0.389
**Vial**	**252**	**1.011**	**0.004**	**2.63** ++	**<0.001**

*Error term used for the test of significance: SS = 1.007, df = 246.321.

**Error term used for the test of significance: SS = 1.007, df = 246.470.

† Error term used for the test of significance: SS = 0.423, df = 262.208.

†† Error term used for the test of significance: SS = 1.007, df = 246.559.

‡ Error term used for the test of significance: SS = 0.425, df = 263.097.

‡‡ Error term used for the test of significance: SS = 0.427, df = 264.103.

+ Error term used for the test of significance: SS = 0.428, df = 264.718.

++ Error term used for the test of significance: SS = 0.337, df = 221.

**Table 2:** Summary of analysis of variance on offspring body size (thorax length) separately for males and females. Significant effects are shown in bold.

Males	df	Type I SS	MS	F	P
**Maternal diet**	**1**	**0.017**	**0.017**	**5.59**	**0.019**
**Paternal diet**	**1**	**0.012**	**0.012**	**4.13**	**0.043**
Maternal diet×Paternal diet	1	0.007	0.007	2.47	0.117
Error	234	0.703	0.003		

Females	df	Type I SS	MS	F	P
**Maternal diet**	**1**	**0.015**	**0.015**	**5.70**	**0.018**
Paternal diet	1	6×10^−5^	6×10^−5^	0.02	0.879
Maternal diet×Paternal diet	1	0.001	0.001	0.53	0.468
Error	239	0.645	0.003		

### Development time

**Figure 3:**
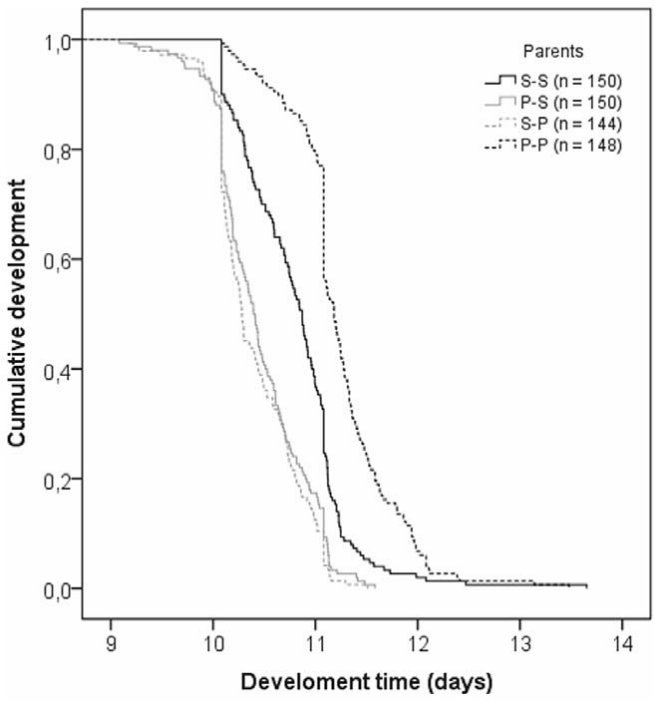
Cumulative development times of offspring (data pooled across sexes). The progeny of P-P parents had the longest development times, those of S-S intermediate development times and those of S-P and P-S parents had the shortest development times. All comparisons were statistically significant except for that between the progeny of S-P and P-S parents. Curves were calculated using the Kaplan-Mayer survival analysis.

Maternal diet, paternal diet, sex and the two-way interaction term paternal diet×maternal diet were included in the model as statistically significant variables predicting development time (Table 3). A significant interaction between the maternal and the paternal diets indicates that a parent's dietary effect on offspring development time was dependent upon the dietary effect of the other parent. According to the results females developed faster than males. Since the effects of parental diet on offspring development time were independent of sex further analyses (Kaplan-Mayer survival analysis) were conducted on data pooled across sexes. It appears that the progeny of P-P parents had the longest development times, those of S-S intermediate development times and those of S-P and P-S parents had the shortest development times (Figure 3). All comparisons were statistically significant except for that between the progeny of S-P and P-S parents (Table 4).

**Table 3:** Development time was analyzed using Cox regression survival analysis.

	OR	Wald	df	P
**Sex**	**0.511**	**63.629**	**1**	**<0.001**
**Maternal diet**	**1.311**	**10.731**	**1**	**0.001**
**Paternal diet**	**1.645**	**35.108**	**1**	**<0.001**
**Maternal diet×Paternal diet**	**0.078**	**192.603**	**1**	**<0.001**
Maternal diet×Sex	1.078	0.208	1	0.649
Paternal diet×Sex	0.867	0.737	1	0.391
Maternal diet×Paternal diet×Sex	1.744	2.818	1	0.093

A significant interaction between the maternal and the paternal diets indicates that a parent's dietary effect on offspring development time was dependent upon the dietary effect of the other parent.

**Table 4:** Kaplan-Meier survival analysis was used for the comparisons of development times.

Parents	x^2</ >^	df	P
S-S vs. P-S	31.732	1	<0.001
S-S vs. S-P	48.764	1	<0.001
S-S vs. P-P	47.839	1	<0.001
P-S vs. S-P	1.446	1	0.229
P-S vs. P-P	160.340	1	<0.001
S-P vs. P-P	191.372	1	<0.001

A reduced probability value of P = 0.05/6 = 0.008 was used to control for multiple comparisons. All comparisons were statistically significant except for that between the progeny of S-P and P-S parents.

Log Rank (Mantel-Cox) statistics are reported.

### Pathogen resistance

Disease treatment and the three-way interactions terms maternal diet×paternal diet×treatment and paternal diet×sex×treatment were included as statistically significant variables in the model (Table 5). According to the results survival was worse among the disease treated flies than among the control flies. To better understand the results binary logistic regression analysis was applied separately for the control and disease treatments. This time, the main effects: parental pair (rearing vial), experiment day, sex, maternal diet and paternal diet, two-way interactions: paternal diet×maternal diet, sex×maternal diet and sex×paternal diet, and a three-way interaction: sex×paternal diet×maternal diet were included as covariates in the model. Among *S. marcescens* infected flies none of the model terms were statistically significant, indicating that disease was the overwhelmingly important factor in survival (Tables 6 and 7).

**Table 5:** Binary logistic regression analysis was used to identify factors associated with pathogen resistance.

	OR	Wald	df	P
Vial		283.372	249	0.066
Maternal diet	8×10^−9^	3×10^−6^	1	0.999
Paternal diet	0.225	1×10^−8^	1	1.000
Sex	1.029	0.032	1	0.858
Experiment day		0.844	2	0.656
**Treatment**	**0.050**	**224.070**	**1**	**<0.001**
Maternal diet×Treatment	0.801	0.310	1	0.578
Paternal diet×Treatment	0.750	0.515	1	0.473
Sex×Treatment	0.892	0.128	1	0.721
**Maternal diet×Paternal diet×Treatment**	**0.037**	**17.115**	**1**	**<0.001**
Maternal diet×Sex×Treatment	0.829	0.158	1	0.691
**Paternal diet×Sex×Treatment**	**3.054**	**5.606**	**1**	**0.018**
Maternal diet×Paternal diet×Sex×Treatment	1.722	0.315	1	0.575

Survival among the disease treated flies was worse than among the control flies.

Overall percentage of cases correctly classified by the model: 86.1%.

Omnibus Tests of Model coefficients: P<0.001.

Hosmer-Lemeshow Goodness of Fit Test: P = 0.039.

Nagelkerke R Square: 0.418.

**Table 6:** Binary logistic regression analysis was used to identify factors associated with pathogen resistance (disease treatment).

Disease-treatment	OR	Wald	df	P
Maternal diet	2×10^−9^	4×10^−6^	1	0.998
Paternal diet	0.349	1×10^−8^	1	1.000
Sex	0.974	0.041	1	0.839
Experiment day		0.873	2	0.646
Vial		192.713	248	0.996
Maternal diet×Paternal diet	0.958	4×10^−12^	1	1.000
Maternal diet×Sex	0.987	0.002	1	0.961
Paternal diet×Sex	1.567	3.022	1	0.082
Maternal diet×Paternal diet×Sex	0.889	0.052	1	0.819

Overall percentage of cases correctly classified by the model: 77.5%.

Omnibus Tests of Model coefficients: P<0.001.

Hosmer-Lemeshow Goodness of Fit Test: P = 0.675.

Nagelkerke R Square: 0.322.

**Table 7:** Binary logistic regression analysis was used to identify factors associated with pathogen resistance (control-treatment).

Control-treatment	OR	Wald	df	P
Maternal diet	1.466	6×10^−10^	1	1.000
Paternal diet	0.928	1×10^−11^	1	1.000
Sex	0.995	1×10^−4^	1	0.992
Experiment day		6×10^−12^	2	1.000
Vial		28.120	248	1.000
Maternal diet×Paternal diet	1.351	9×10^−11^	1	1.000
Maternal diet×Sex	1.309	0.066	1	0.797
Paternal diet×Sex	0.055	7.724	1	0.005
Maternal diet×Paternal diet×Sex	0.084	1.407	1	0.236

Overall percentage of cases correctly classified by the model: 97.4%.

Omnibus Tests of Model coefficients: P<0.001.

Hosmer-Lemeshow Goodness of Fit Test: P = 0.781.

Nagelkerke R Square: 0.686.

## Discussion

According to our results offspring whose mothers were raised on poor food as larvae were larger than offspring whose mothers were raised on standard food. Furthermore, male progeny sired by fathers that were raised on poor food were larger than male progeny sired by males raised on standard food. No effect of paternal diet on adult body size of the female offspring was detected. Egg-to-adult development times were shortest for offspring whose one parent was raised on standard and the other parent on poor food (P-S, S-P). Offspring whose parents were raised on standard food (S-S) had intermediate development times. The longest development times were found among offspring whose parents both had experienced poor nutritional conditions as larvae (P-P). No evidence for transgenerational effects of parental nutrition on offspring disease resistance was found.

### Transgenerational effects of parental early nutrition

According to life-history theory natural selection could be expected to favor parents that produce fewer but better provisioned offspring in response to cues indicative that offspring will experience nutritional stress [63], [64]. In organisms that lack parental care, egg or newborn size can be used as an estimate of parental provisioning [65]. Two studies have previously investigated the effect of maternal diet on offspring performance in *D. melanogaster* [6], [23]. According to Vijendravarma et al. [6] *D. melanogaster* females raised on poor larval food laid heavier eggs than females raised on standard food which, according to the authors, could indicate enhanced egg provisioning by poorly fed mothers. Moreover, offspring raised on poor food were found to develop faster and be lighter if their mothers also developed on poor food. The extent to which the faster development of offspring of parents raised on poor food was due to the larger egg size rather than to maternal effects mediated otherwise was not determined. It was suggested by the authors that although maternal history of poor nutrition may have adverse effects on some traits, adaptive plastic responses on other traits may act to alleviate these negative effects. For some traits the plastic response may even be strong enough for the offspring of the poorly-fed mothers to perform better under poor nutritional conditions than the offspring of well-fed mothers [6]. In contrast to our results, no effect of maternal diet on development time and body size was detected when the offspring were raised on standard food [6]. In a study by Prasad et al. [23] poorly nourished *D. melanogaster* mothers also showed a tendency of laying heavier eggs than well fed mothers, however, no effect of maternal diet on offspring dry weight at eclosion was observed. Although paternal effects have been previously demonstrated in *D. melanogaster* [45]–[48] no earlier studies have investigated male-mediated transgenerational effects of diet in this species. In the fly *T. angusticollis* (also a species in which there is no evidence of paternal provisioning) variation in the larval diet quality has however been shown to be transmitted across generations through maternal and paternal effects [24]. In this species both mothers and fathers were found to transfer their condition to their offspring, but with effects on different offspring traits [24].

Azevedo et al. [66] studied the effects of egg size on offspring fitness components in *D. melanogaster* and found that although egg size had a positive effect on hatchling weight and development time it had no consistent effects on adult weight. In the light of the above mentioned studies in *D. melanogaster* it appears unlikely that the observed larger adult size of offspring born to mothers raised on poor larval food would be the result of an enhanced egg provisioning by poorly fed mothers. Moreover, since in insects, including *D. melanogaster*, larger egg size is typically associated with shorter development [66], the observed faster development of offspring born to parents raised on standard food would indicate enhanced egg provisioning by parents raised on standard food. This would, however, be in contrast to what previous studies in this species have reported (see above) [6], [23]. Moreover, it would not be consistent with the adaptive response predicted by life-history theory [63], [64]. On the other hand, since it is believed that the evolution of larval growth rate and adult body is shaped by the tradeoff between the fitness benefits of being large versus those of developing to adulthood fast [65], [67]–[69], it is possible that the larger size of offspring whose parents were raised on poor food reflects a trade-off with the slower development of these offspring (i.e. due to the slow development of offspring of P-P parents). Hence, by directly affecting one of the two traits, development time or adult size, parental nutrition could have caused indirect changes in the other trait.

Our results are similar to those reported by Vijendravarma et al. [6] in that parental dietary effects would seem to involve both adaptive as well as maladaptive effects on offspring performance. According to the results of our study dietary effects of both mothers and fathers can however be transmitted to the next generation and, such effects can be found when the offspring are raised on standard food. The results of the present study could suggest that under appropriate nutritional conditions an individual's life-history strategy may, at least to some extent, be determined by the nutritional history of its parents. Consequently, when raised under standard nutritional conditions offspring whose parents were raised on standard food would develop faster but be smaller as adults than offspring whose parents were raised on poor food; offspring whose parents have a history of malnourishment would have the opposite strategy. Which of the two life-history strategies is most beneficial under the given circumstances cannot be identified by our experimental setup.

### Possible fitness consequences of parental effects

By comparing development times of offspring of P-P parents with those of S-S parents it would appear that parents transferred their condition to their offspring. However, because the shortest development times were found among offspring whose one parent was raised on standard and the other parent on poor food (P-S, S-P) the mechanistic basis appear more complicated than that. Although the fitness benefits of developing to adulthood fast may be more apparent in the wild where the larval food sources of *D. melanogaster* (decaying fruit) are likely to become unsuitable over time, the larval nutritional environment in the laboratory is also likely to deteriorate with time as the resources are used up by competing larvae and due to the accumulation of waste products. Parental effects on offspring performance have been suggested to be most important when poor environmental conditions are encountered by juveniles [6], [24], [70]. Being able to develop fast could indeed be particularly advantageous when larvae are developing under adverse nutritional conditions, where development is generally slow [6].

The fitness benefits of developing to adulthood fast and those of being large often trade off with each other [65], [67]–[69]. Whether the observed parental effects on offspring size are sufficient to affect offspring fitness was not determined by us. Because in invertebrates, including *D. melanogaster*, body size is often positively correlated with female fecundity and male mating success [67]–[69], there are strong grounds for suspecting that regardless of their slower development offspring of parents raised on poor diet would have some fitness advantages due to their larger size. According to Monaghan [71] phenotypic changes that take place during development in response to environmental cues but where the advantage of the induced phenotype is not apparent until later in life should not however be costly in the juvenile stages, otherwise they would be selected against because the forces of selection are likely to be stronger in the younger stages. If the life-history strategies determined by parental effects are fixed, the advantages of adopting a particular life-history strategy will most probably depend on the prevailing environmental conditions. Further studies investigating parental effects under a full set of environmental crossovers between parental and offspring environments are needed to reveal whether parental nutrition really sets patterns of resource allocation in the offspring and whether such effects are sufficient to limit the offspring's ability to respond to new conditions.

### Paternal effects and the evolution of female mate choice

In the present study both maternal and paternal dietary effects on offspring size were detected. Whereas the effect of maternal nutrition on offspring size was independent of sex, paternal diet only affected the size of the male offspring. In *D. melanogaster* the advantage of larger males in competition for mates is rather well documented [72]–[74]. In species lacking conventional forms of paternal provisioning sire effects have been implicated an interesting role in the evolution of female mating preferences [24]. When females prefer to mate with ‘attractive’ males (often those with elaborate secondary-sexual characteristics) but do not receive direct benefits from their mate-choice behavior, it is surmised that females gain indirect genetic benefits from their choice [75]. Over time, persistent female preference for attractive males should however erode genetic variance in the characteristics that the female preference is based upon and eventually, the benefits associated with the preferences would be lost. Nonetheless, female preferences for these traits seem to persist in many taxa [75]. Since purely environmental variation will continue to affect phenotypically plastic traits regardless of genetic variance, it has been suggested that if environmental variation in paternal condition could be transmitted to offspring through paternal effects it could contribute to indirect selection on female preferences [4].

In the fly *T. angusticollis*, in which a paternal diet effect on offspring body size was observed (see above), large, high condition fathers were found to produce larger offspring and it was shown that this paternal effect was sufficient to increase mating success of male offspring and fecundity of female offspring [24]. Although in the present study maternal effects were somewhat more important than paternal effects in explaining variation in male body size (Table 2), males raised under poor nutritional conditions were found to sire larger sons than males raised on standard food. Whether these effects are sufficient to affect male mating success was not determined. The role of paternal effects, if any, in the evolution of female mating preferences and determining male mating success in *D. melanogaster* needs further investigation.

### Possible mechanisms for the transfer of paternal effects in *D. melanogaster*

While our study demonstrates the importance of not only considering the relative contributions each parental sex has on progeny performance but also the potential interactions that may exist among the sexes it does not address the underlying modes of action. In general, whereas maternal effects comprise a number of phenomena [2], [76] the possible factors contributing to paternal effects are less clear. In a study by Giesel et al. [45] the effects of maternal and paternal photoperiod on progeny development time were found to be roughly equal in *D. melanogaster*. According to the authors the effect of paternal photoperiod could only be due to alterations in the character of nuclear genomic information since passage of cytoplasmic elements to progeny via sperm is not known to occur in this species.

*D. melanogaster* has a promiscuous mating system and no parental care. In this species males and females only interact during courtship and copulation. With notable exceptions [48], [77], egg volume and size are considered to be determined solely by the maternal genotype in *D. melanogaster* [66]. A male mediated effect of temperature on egg size has however been demonstrated in this species [48, but see 43]. In addition, a recently published paper by Pischedda et al. [77] demonstrates that male *D. melanogaster* vary genetically in their influence on egg size. Although these studies did not identify the underlying mechanistic bases for the observed paternal effects, it was suggested by Pischedda et al. [77] that differential female investment in reproduction based on the perceived quality of the mate or alternatively, variation in the ability of males to manipulate female reproductive investment could explain the results. If variation is directly caused by males, it could, according to Pischedda et al. [77], occur via variation in male seminal proteins [77]–[79]. In *D. melanogaster* the entire sperm is incorporated into the egg during fertilization and may have functional relevance in the early development [80]. Moreover, genes carried by the sperm, the so called paternal effect genes, work during fertilization and are essential for zygote formation and viability [81]. At mating *D. melanogaster* males transfer both sperm and a cocktail of seminal fluid proteins (Sfps) to their mates [78], [79]. According to Markow and Ankney [44] incorporation of nutrients from the male ejaculate does not occur in this species. Although Sfps are rather well characterized in *D. melanogaster* the full set of proteins transferred to females, let alone their functions, have not been defined [79]. In some invertebrate species the accessory gland proteins, the major components of *D. melanogaster* Sfps, have been suggested a role in mediating transgenerational parental effects [38]–[40]. According to a study by Fricke et al. [82] the magnitude of female *D. melanogaster* response to a specific ejaculate component, the sex peptide, is significantly affected by the nutritional environment (variation in the amount of yeast provided) in which adult females are maintained. Hence, paternal effects if they are proven to occur via seminal proteins in *D. melanogaster* could, at least in theory, also be affected by nutritional conditions experienced by the parents. Moreover, epigenetic modifications of sperm DNA could play a role in paternal transmission of dietary effects [41], [42]. In a recently published paper by Friberg et al. [43] transgenerational epigenetic effects were indeed considered the most feasible candidate for the paternal effects on egg-to-adult survival found in *D. melanogaster*. The occurrence of paternal effects in species where there is no paternal care suggests that the fertilizing sperm has more function than hitherto thought [38]–[41], [45]–[48], [81].

### Conclusions

Past environmental conditions, especially those experienced by the mother, are considered important in shaping offspring phenotype, moreover, they have been shown to play an important role in determining the way offspring respond to current environmental conditions [6], [17], [22]–[26]. The extent to which maternal environment influences offspring phenotype and fitness is considered to determine whether such effects themselves will be acted on by natural selection [2]. The existence of paternal effects indicates that paternal experience may also be translated into variation in offspring fitness. In addition to their practical significance such effects would have important theoretical implications in the field of quantitative genetics for their potential to inflate estimates of additive genetic variance [43]. The emerging evidence supporting the occurrence of paternal effects in species with no paternal care suggests that such effects are far more common than hitherto appreciated. Whether parental effects are independent of the mate, or whether parental effects generally change depending on the combination of the parental phenotypes need further investigation. In order to be able to generalize, this work must include species from multiple taxa.

In conclusion, this work highlights the importance of not only considering the relative contribution each parental sex has on progeny performance but also the possibility of their joint effects. Furthermore, the results of the present study suggest that under appropriate nutritional conditions an individual's life-history strategy may be set by the nutritional history of its parents. Further work is required to investigate whether such life-history strategies are fixed or can be overcome with a change in diet. In the present study, we studied the relationship between parental early nutrition and one component of invertebrate immunity and observed no effect. Since different components of the immune system do not necessarily show correlated responses [83], it would be of interest to investigate the effect of parental early nutrition on other aspects of offspring immunity.
